# Mechanism of luteolin against non-small-cell lung cancer: a study based on network pharmacology, molecular docking, molecular dynamics simulation, and *in vitro* experiments

**DOI:** 10.3389/fonc.2024.1471109

**Published:** 2024-11-08

**Authors:** Jihang Zhang, Changling Li, Wenyi Li, Zhenpeng Shi, Zhenguo Liu, Junyu Zhou, Jing Tang, Zixuan Ren, Yun Qiao, Deshan Liu

**Affiliations:** ^1^ The First Clinical Medical College, Shandong University of Traditional Chinese Medicine, Jinan, China; ^2^ Research Center for Basic Medical Sciences, Qilu Hospital of Shandong University, Jinan, China; ^3^ Department of Traditional Chinese Medicine, Qilu Hospital of Shandong University, Jinan, China

**Keywords:** luteolin, network pharmacology, non-small-cell lung cancer, Akt/MDM2/p53 signaling pathway, *in vitro* experiments

## Abstract

**Introduction:**

Luteolin, a naturally occurring flavonoid compound, demonstrates promising anti-cancer properties. However, its mechanism against non-small-cell lung cancer (NSCLC) remains unknown. This study employed network pharmacology, molecular docking, molecular dynamics simulation (MDS), and *in vitro* experiments to investigate the potential mechanisms by which luteolin against NSCLC.

**Methods:**

Initially, the potential targets of luteolin and NSCLC-related targets were identified from public databases such as TCMSP, GeneCards, OMIM, DrugBank, and TTD. Subsequently, the protein-protein interaction (PPI) network screening and Gene Ontology (GO) and Kyoto Encyclopedia of Genes and Genomes (KEGG) enrichment analyses were conducted. The binding affinity and stability of luteolin with the core targets were assessed using molecular docking and MDS. Finally, the results were validated by *in vitro* experiments.

**Results:**

A total of 56 luteolin targets and 2145 NSCLC-related targets were identified. Six core targets, TP53, EGFR, AKT1, TNF, JUN, and CASP3, were screened via the PPI network. The GO and KEGG analyses indicated that luteolin’s activity against NSCLC potentially involves PI3K-Akt, NF-kappa B, and other signaling pathways. Molecular docking revealed that luteolin had high binding affinity with the core targets. MDS confirmed the stable interaction between luteolin and key proteins TP53 and AKT1. *in vitro*, luteolin significantly inhibited the proliferation and migration of A549 cells, while also inducing apoptosis. In addition, luteolin downregulated the expression of p-Akt (Ser473), MDM2, and Bcl-2 but upregulated the expression of p53 and Bax, which was consistent with the effect of LY294002.

**Conclusion:**

Luteolin had a good anti-NSCLC effect, and the apoptosis-inducing effect might be related to the Akt/MDM2/p53 signaling pathway.

## Introduction

Lung cancer is one of the most common cancers and a leading cause of cancer-related mortality worldwide (1, 2). According to GLOBOCAN, there were almost 2.5 million new cases and 1.8 million deaths from lung cancer worldwide in 2022 (3). Lung cancer is categorized into two broad histological subtypes: small-cell lung cancer and non-small-cell lung cancer, with NSCLC accounting for approximately 85% of all cases (4, 5). As the primary histological subtype of lung cancer, NSCLC represents a significant threat to human health. Currently, the main treatment options for NSCLC include surgical resection, radiotherapy, chemotherapy, immunotherapy, and molecular targeted therapy (6). However, these methods may not fully meet the expectations. For instance, surgical resection alone is not curative for many patients with early-stage NSCLC, and the risks of recurrence and metastasis increase with higher stage (7). Moreover, most conventional chemotherapeutic drugs exhibit the same limitations, such as non-specific targeting, low bioavailability, and drug resistance (8). Compared to conventional drugs, molecular targeted therapy can selectively kill cancer cells and possess fewer side effects, but there is also drug resistance.

Pleiotropic natural products represent a promising strategy for cancer treatment due to their multi-target effects and low toxicity (9, 10). Approximately 80% of approved chemotherapeutic drugs and over half of all pharmaceuticals are derived from natural products such as paclitaxel, vincristine, and adriamycin (11). Luteolin (3,4,5,7-tetrahydroxy flavone), a natural flavonoid found in fruits, vegetables, and herbs, exhibits multiple biological activities including anti-inflammation, anti-oxidation, anti-allergy, anti-cancer, and immunoregulatory (12, 13). Of these, luteolin exhibits potent inhibitory effects against a diverse range of malignant tumors, including breast cancer (14), pancreatic cancer (15), prostate cancer (16), colon cancer (17), and lung cancer (18). For example, luteolin has been shown to inhibit the stemness of breast cancer through the Nrf2-mediated pathway and to enhance chemosensitivity in combination with paclitaxel (19). Research by Jiang et al. demonstrated that luteolin suppressed proliferation, induced apoptosis, and decreased PD-L1 expression in lung cancer with KRAS-mutantion (20). Another study indicated that luteolin reversed epithelial-mesenchymal transition (EMT) by suppressing the Notch signaling pathway (21). However, the mechanism of luteolin against NSCLC remains unclear.

Network pharmacology, first introduced by the British pharmacologist Hopskin, is regarded as a novel interdisciplinary domain of study (22). It can construct complicated pharmacology networks based on compounds, biological functions, and target proteins, which is consistent with the overall feature of natural products and is not available in conventional studies (23). The integration of network pharmacology, molecular docking and experimental validation has been widely used to study potential anticancer compounds (24–26). In this study, network pharmacology, molecular docking, molecular dynamics simulation, and *in vitro* experiments were employed to elucidate the mechanisms of luteolin against NSCLC, providing a reference for further research and application of luteolin.

## Materials and methods

### Targets prediction of luteolin

The keyword “luteolin” was queried in the Traditional Chinese Medicine Systems Pharmacology Database and Analysis Platform (TCMSP, https://tcmspw.com/index.php), and the relevant targets identified from the search were utilized as the predicted targets of the compound. Subsequently, the target information was entered into the UniProt database (http://www.uniprot.org/) to retrieve the corresponding gene names.

### Targets prediction of NSCLC

The GeneCards database was utilized to identify NSCLC-related targets using a screening criterion of a relative score ≥ 2-fold median. Additionally, we also performed a search in OMIM (http://www.omim.org), DrugBank (https://go.drugbank.com), and TTD (http://db.idrblab.net/ttd) to identify the disease-related targets. The data retrieved from these databases were subsequently merged.

### Construction of protein-protein interaction network

The overlapping targets between luteolin and NSCLC were identified using the jvenn database (http://bioinfo.genotoul.fr/jvenn). These common targets were then input into the STRING database (https://string-db.org/) to construct a PPI network. The “organism” parameter was set to “Homo sapiens,” and the minimum required interaction score was established at greater than 0.4. The result was then imported into Cytoscape_v3.7.2 software for visualization. The “Analyze Network” tool in the Cytoscape software was employed to calculate the degree values of the nodes. Subsequently, nodes with high degree values were selected as the core targets.

### GO and KEGG enrichment analysis

Gene Ontology (GO) and Kyoto Encyclopedia of Genes and Genomes (KEGG) enrichment analyses of common targets were conducted using the Metascape database (http://metascape.org/). Statistical significance was established at *P*<0.01. Subsequently, we utilized the bioinformatics platform (http://www.bioinformatics.com.cn/) to generate a KEGG enrichment bubble diagram and a GO enrichment bar diagram.

### Construction of Drug-Target-Pathway-Disease network

To analyze the complex associations among luteolin, overlapping targets, related pathways, and NSCLC, we constructed a Drug-Target-Pathway-Disease network using Cytoscape_v3.7.2 software.

### Molecular docking

Molecular docking technology was utilized to analyze the interactions between luteolin and its core targets. The crystal structures of TP53 (PDB ID: 6gge), EGFR (PDB ID: 8a27), AKT1 (PDB ID: 1unq), TNF (PDB ID: 5uui), JUN (PDB ID: 1jnm), CASP3 (PDB ID: 1nme) were obtained from the RCSB Protein Data Bank (PDB, https://www.rcsb.org/), and the MOL2 (3D) format file of luteolin was downloaded from the TCMSP datebase. Subsequently, the proteins were processed using PyMOL 2.3.0 to remove water molecules, co-crystallized ligands, irrelevant protein chains, and ions (27). Thereafter, drug and protein files were imported into AutoDock Tools 1.5.7 to separate the proteins, add non-polar hydrogens and calculate the Gasteiger charges before being saved them in PDBQT format (28). The target proteins were receptors, and luteolin was ligand. The grid box for docking was constructed around the geometric center, based on the position of the original ligands, and adjusted to ensure coverage of the docking pockets. Molecular docking and affinity calculations were performed utilizing AutoDock Vina 1.1.2 (29). The lower the binding energy, the greater the affinity between the ligand and its receptors. The conformation exhibiting the best affinity was selected as the final docking conformation and visualized using PyMOL software.

### Molecular dynamics simulation

All-atom molecular dynamics simulations were performed using the docked complexes as initial structures, and the simulations were performed using GROMACS v.2022 (30). The AMBER force field was used to describe the proteins. The pdb2gmx subprogram was used to add hydrogen atoms to the system, a truncated cubic TIP3P solvent box was added to the system at a distance of 10 Å (31), and Na+/Cl- was added to the system for balancing the system charge, and finally the topology and parameter files used for the simulations were outputted. Before the simulations, energy minimization was performed using the “mdrun” command and steepest descent method (canonical system synthesis), with the starting step set to 0.01 nm and a maximum force tolerance of 1000 kJ/mol·nm. After the energy minimization, a 100 ps NVT (isothermal-isobaric) ensemble simulation at a fixed volume and constant rate of temperature increase was used to slowly increase the temperature from 0 K to 310 K. A 100 ps NPT (isobaric-isobaric) ensemble simulation was then performed using the Berendsen barostat to equilibrate the pressure of the solvent with the complex system to 1 bar. During MDS, hydrogen bonds were constrained using the LINCS algorithm with an integration step of 2 fs. The electrostatic interactions were calculated using the Particle-mesh Ewald (PME) method with a cutoff of 1.2 nm. The cutoff for non-bonded interaction was set to 10 Å and updated every 10 steps.

### Cell culture

The human p53 wild-type (wt) NSCLC cell lines A549 and H460 were procured from Procell Life Science & Technology Co., Ltd. Both cell lines were cultured in RPMI-1640 medium (Procell, China) supplemented with 10% fetal bovine serum (FBS, Procell, China), penicillin (100 U/ml, Gibco, USA), and streptomycin (100 μg/ml, Gibco, USA). The cultures were maintained at 37°C in a 5% CO_2_ atmosphere.

### Cell viability assay

A549 and H460 cells were seeded into 96-well plates at a density of 2×10^3^ cells per well and subsequently treated with various concentrations of luteolin (0, 10, 20, 40, 60, 80 μM) for 24, 48, or 72 hours. After treatment, 10 μL of CCK-8 solution (Glpbio, USA) was added to each well and incubated for an additional hour. Absorbance was measured at 450 nm using a microplate reader (Tecan, Switzerland).

### Colony formation assay

A549 cells were seeded in 6-well plates at a density of 800 cells per well and subsequently treated with different concentrations of luteolin (0, 20, 40, 60 μM) for one week, with media refreshments every three days.

At the end of treatment, cells were fixed using 4% paraformaldehyde (Biosharp, China) for 15 minutes, stained with crystal violet staining solution (Beyotime, China) for 10 minutes, and left to dry overnight. Colonies were then counted using an inverted microscope (Nexcope, China). A cluster was defined as a colony if it contained 50 or more cells.

### Wound healing assay

A549 cells were seeded in 6-well plates until they reached 80% confluence. A wound was made at the bottom using a 200-μl pipette tip. The plates were then washed twice with PBS, and the cells were treated with luteolin (0, 20, 40, 60 μM). The scratches at 0 h and 24 h were observed using an inverted microscope. The wound area was quantified with Image J software.

### Hoechst 33342 staining

A549 cells were seeded in 6-well plates at a density of 10 × 10^4^ cells per well and subsequently treated with luteolin (0, 20, 40, 60 μM) and LY294002 for 48 hours. After the treatment, the cells were stained with Hoechst 33342 staining solution (Beyotime, China) for 15 minutes and imaged under a fluorescence microscope (Nikon, Japan).

### Flow cytometry analysis

A549 cells were seeded in 6-well plates at a density of 10 × 10^4^ cells per well and subsequently treated with luteolin (0, 20, 40, 60 μM) and LY294002 (CST, USA) for 48 hours. After the treatment, the cells were digested using a trypsin solution without EDTA (Solarbio, China). Following collection, the cells were stained using an Annexin V-PI apoptosis detection kit (Vazyme Biotech, China) and apoptosis was assessed via flow cytometry (BD Biosciences, USA).

### Western blot

Total cellular protein was extracted using RIPA lysis buffer (Servicebio, China). Protein concentrations were measured with a BCA protein assay kit (Share-bio, China). Equal amounts of proteins were separated using 12.5% SDS-PAGE gel electrophoresis (Sangon Biotech, China) and subsequently transferred to PVDF membranes (Millipore, USA). The membranes were then blocked with 5% BSA (Solarbio, China) for 2 hours at room temperature and incubated overnight at 4°C with primary antibodies. Following this, the membranes were washed with TBST buffer (Solarbio, China) and incubated for 1 hour at room temperature with secondary antibodies. Finally, the target proteins were visualized with an ECL kit (Glpbio, USA), and the band gray values were analyzed using Image J software.

The primary antibodies used in these experiments included: anti-Akt (Cat No: WL0003b, Wanleibio, China), anti-phospho-Akt (Cat No: 4060, CST, USA), anti-MDM2 (Cat No: TA801705S, OriGene, USA), anti-p53 (Cat No: ab179477, Abcam, USA), anti-Bcl-2 (Cat No: ab32124, Abcam, USA), anti-Bax (Cat No: TA810334S, OriGene, USA), and anti-β-actin (Cat No: 81115-1-RR, Proteintech, USA).

### Statistical analysis

GraphPad Prism 8.0 software was used for statistical analysis of the results. All results were expressed as mean ± standard error (SE) of three independent experiments. Comparisons between groups were performed by one-way analysis of variance (ANOVA). The difference between the groups was considered statistically significant if the *P*-value was less than 0.05.

## Results

### Potential targets of luteolin

A total of 56 potential targets of luteolin were identified using the TCMSP database, and their corresponding gene names were retrieved through the UniProt database (**Supplementary Table S1**).

### Related targets of NSCLC

We conducted a search in the GeneCards database for targets related to NSCLC and calculated the median relative score. Subsequently, we isolated 1662 targets with a relative score ≥ 31.33. Moreover, we retrieved 468 targets from the OMIM database, 53 from the DrugBank database, and 102 from the TTD database. After eliminating duplicates, we identified 2,145 relevant targets for NSCLC (**Supplementary Table S2**).

### PPI network

**Figure 1:**
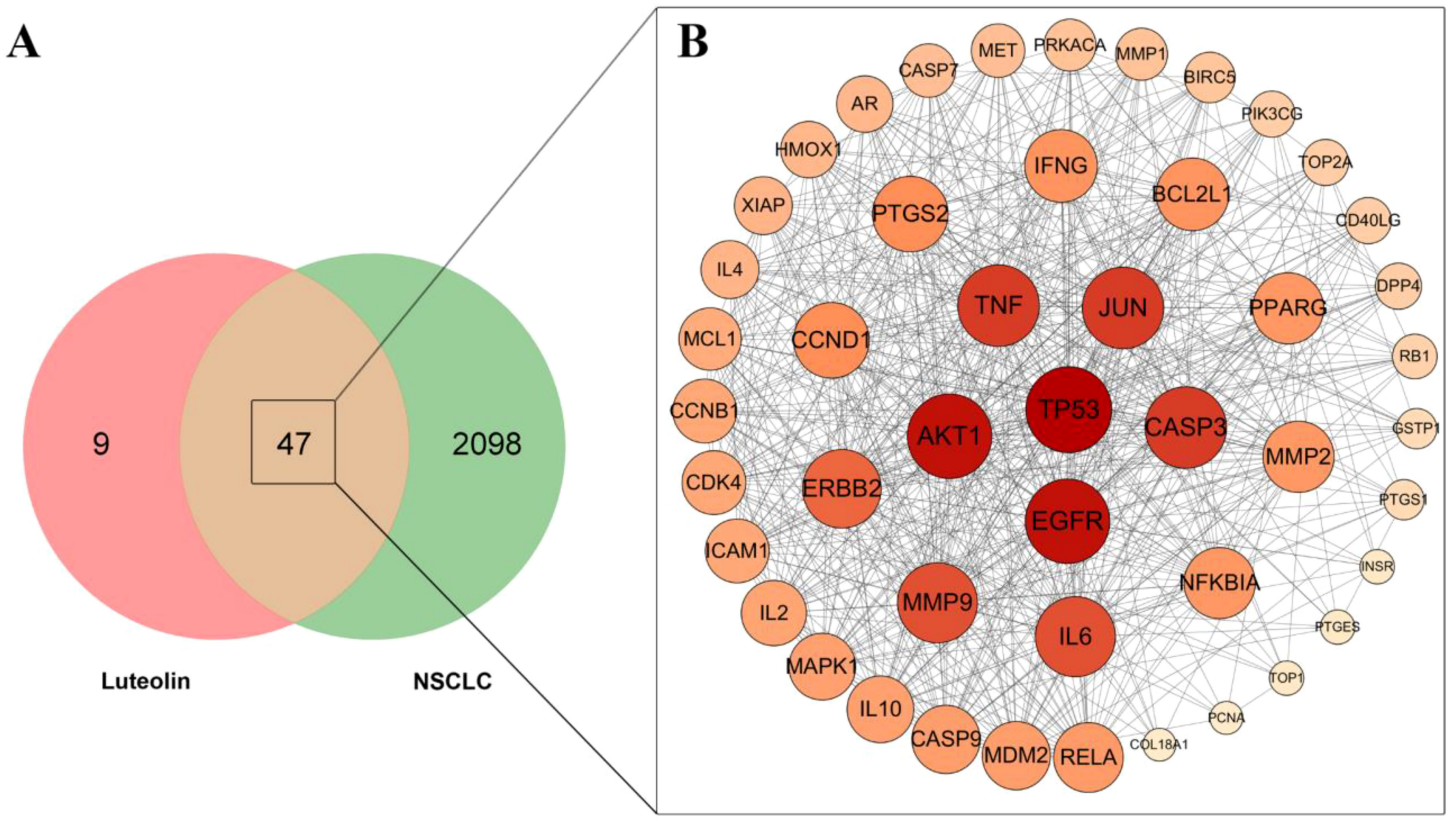
Venn diagram and PPI network analysis. **(A)** Venn diagram of the overlapping targets. **(B)** PPI network of the common targets of luteolin and NSCLC. The size and color depth of the nodes is proportional to their degree values.

The jvenn database was ultilized to identify overlapping targets, resulting in 47 common targets (**Figure 1A**). These common targets were then imported into the STRING database to construct a PPI network, subsequently visualized using Cytoscape_v3.7.2 (**Figure 1B**). The targets with the highest degree values, specifically TP53, EGFR, AKT1, TNF, JUN, and CASP3, were identified as core targets.

### GO and KEGG enrichment analysis

**Figure 2:**
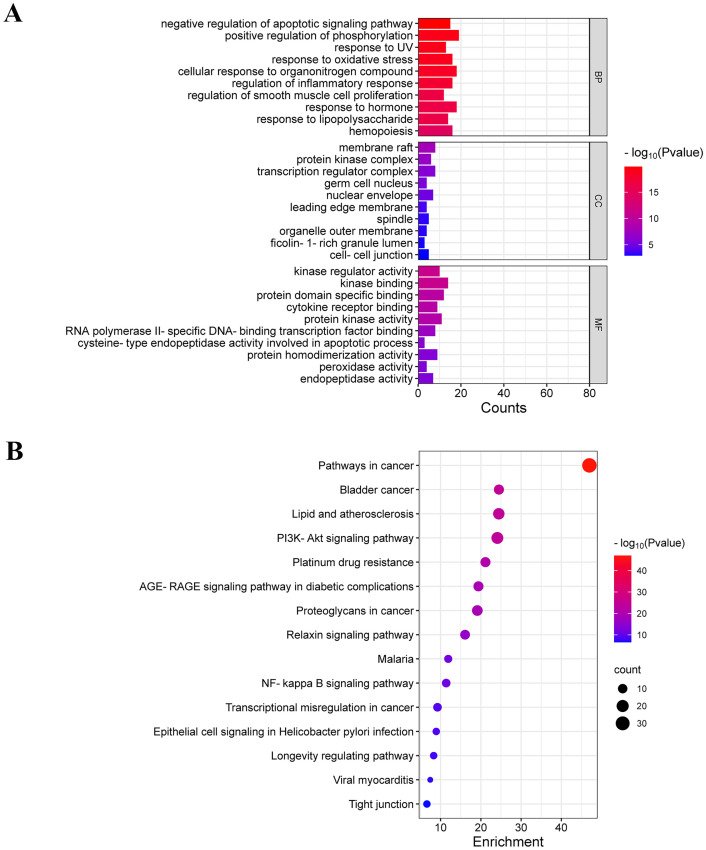
GO and KEGG enrichment analysis. **(A)** GO enrichment analysis of common targets (top 10 were listed). **(B)** KEGG pathways enrichment analysis of common targets (top 15 were listed).

GO and KEGG enrichment analyses were conducted on common targets using the Metascape database. The top 10 enriched terms from biological process (BP), cellular component (CC), and molecular function (MF) were visualized in a bar diagram (**Figure 2A**). BP terms primarily involved negative regulation of apoptotic signaling pathway, positive regulation of phosphorylation, response to UV, and response to oxidative stress. CC terms mainly included membrane raft, protein kinase complex, and transcription regular complex. MF terms mainly included kinase regulator activity, kinase binding, and protein domain specific binding.

The top 15 enriched KEGG terms were visualized in a bubble diagram (**Figure 2B**). KEGG enrichment analysis indicated that luteolin against NSCLC via the PI3K-Akt signaling pathway, proteoglycans in cancer, NF-kappa B signaling pathway, and transcriptional misregulation in cancer. The PI3K-AKT pathway is a pivotal signaling pathway in the progression of lung cancer. According to the PPI network, AKT and TP53 are core targets of luteolin in the treatment of NSCLC. TP53, a vital oncogene, is crucial in inducing apoptosis in lung cancer cells. It has been found that AKT mediates the degradation of wild-type p53 by activating the ubiquitin ligase MDM2 (32, 33). Notably, MDM2 is a common target for both luteolin and NSCLC. Therefore, we hypothesized that luteolin maight trigger apoptosis by inhibiting the Akt/MDM2/P53 pathway.

### Drug-Target-Pathway-Disease network

**Figure 3:**
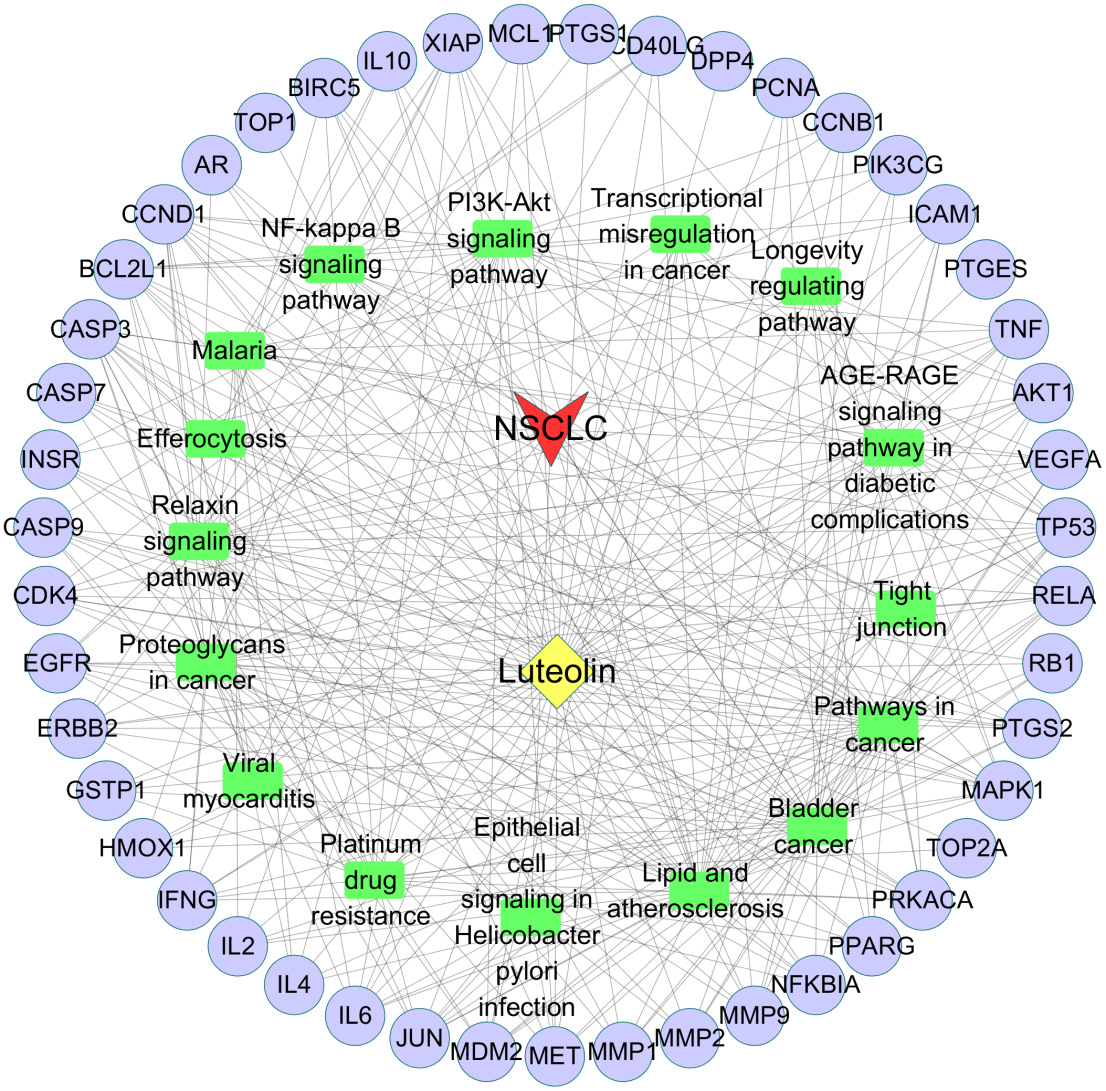
Drug-Target-Pathway-Disease network diagram. Purple nodes represent the common targets. Green rectangles represent the signaling pathways. Yellow diamond represents luteolin. Red arrow represents NSCLC.

Utilizing Cytoscape v3.7.2 software, a Drug-Target-Pathway-Disease network diagram was constructed, as shown in **Figure 3**.

### Molecular docking

**Figure 4:**
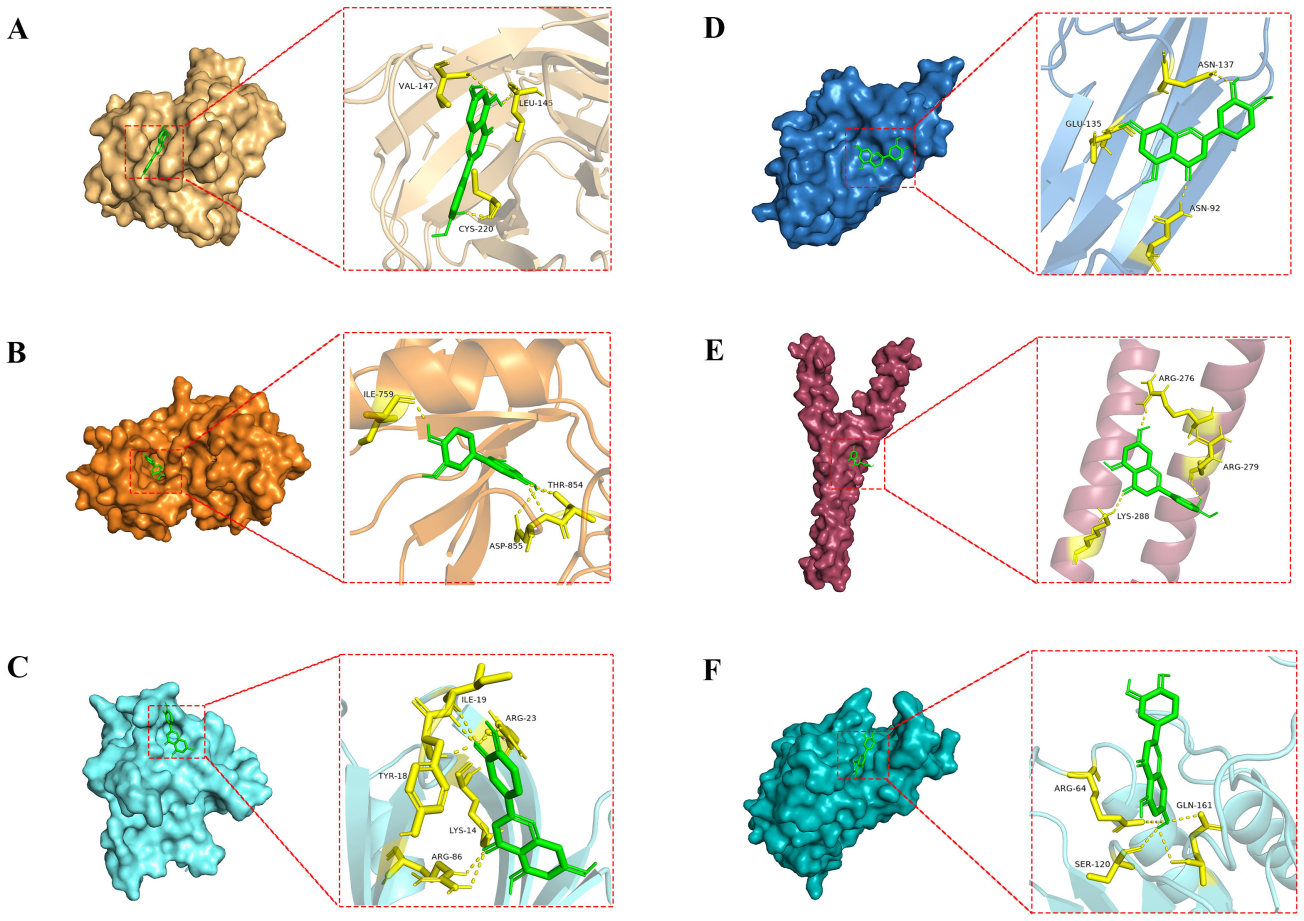
Molecular docking modes of luteolin with the core targets. **(A)** luteolin-TP53, **(B)** luteolin-EGFR, **(C)** luteolin-AKT1, **(D)** luteolin-TNF, **(E)** luteolin-JUN, and **(F)** luteolin-CASP3.

To investigate the docking mode and binding affinity of luteolin with the core targets, molecular docking was conducted. As shown in **Table 1**, the binding affinity of the core targets with luteolin was all less than -5.0 kcal/mol, indicating these targets could stably bind with luteolin (34, 35). The molecular docking modes are shown in **Figure 4**. These findings demonstrated the reliability of network pharmacological results.

**Table 1:** The docking information of the core targets with luteolin.

Targets	PDB ID	Grid Box Center (x, y, z)	Affinity (kcal/mol)
TP53	6gge	(91.29, 95.207, -44.555)	-7.1
EGFR	8a27	(24.621, -10.003, -13.829)	-9.2
AKT1	1unq	(15.18, 24.427, 16.345)	-5.9
TNF	5uui	(41.438, 43.124, 1.354)	-5.4
JUN	1jnm	(10.22, 0.513, 29.418)	-5.7
CASP3	1nme	(36.18, 93.474, 18.309)	-7.5

### Molecular dynamics simulation

**Figure 5:**
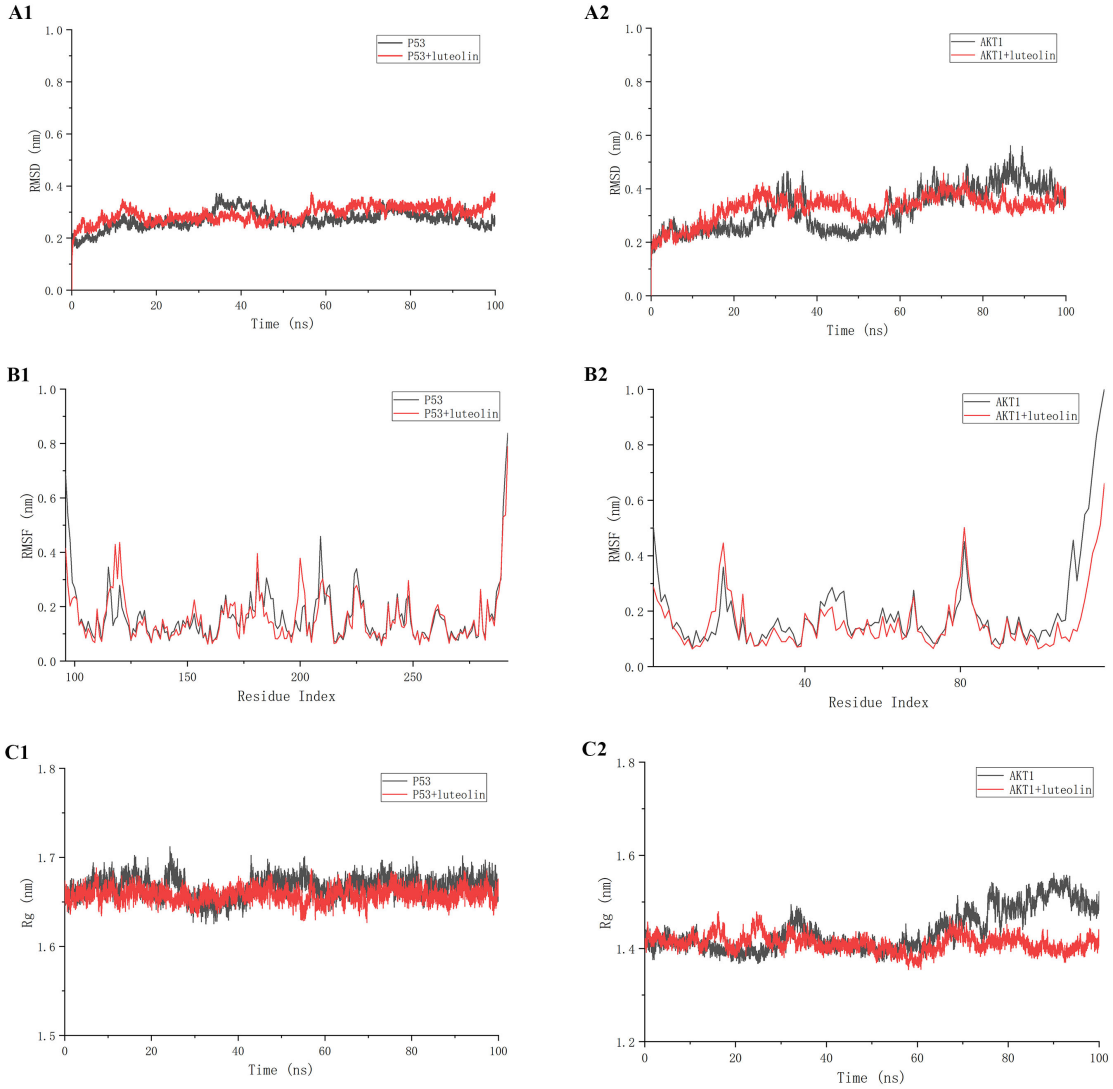
The results of molecular dynamics simulation. **(A1, A2)** Root Mean Square Deviation (RMSD) values. **(B1, B2)** Root Mean Square Fluctuation (RMSF) values during molecular dynamics simulations. **(C1, C2)** Radius of Gyration (Rg) analysis.

Prior to *in vitro* validation, we investigated the stability and structural changes of luteolin binding to core proteins within the AKT/MDM2/P53 signaling pathway through molecular dynamics simulations. Following the molecular docking results, simulations of both luteolin-TP53 and luteolin-AKT1 were conducted for 500 ns. The Root Mean Square Deviation (RMSD) is commonly employed to assess the extent of structural variations from the initial molecular structure. As shown in **Figures 5A1, A2**, the RMSD values of both the luteolin-P53 system and the luteolin-AKT1 system were stabilized after 20 ns, indicating that the binding of luteolin to P53 and AKT1 was stable. The Root Mean Square Fluctuation (RMSF) is commonly used to represent the degree of fluctuation of individual atoms in molecules. As shown in **Figures 5B1, B2**, the RMSF values of amino acid residues in the luteolin-P53 system (except the residues 117~124, 150~157, 168~172, and 198~203) were lower than those in the P53 system. Similarly, the RMSF values of residues in the luteolin-AKT1 system (except the residues 14~23 and 78~83) were lower than those in the AKT1 system. The Radius of Gyration (Rg) is used to identify the compactness of molecules. As shown in **Figures 5C1, C2**, the fluctuation ranges of the Rg values of both the luteolin-TP53 system and the luteolin-AKT1 system were small throughout the simulations.

### Luteolin inhibited the proliferation of A549 cells and H460 cells

**Figure 6:**
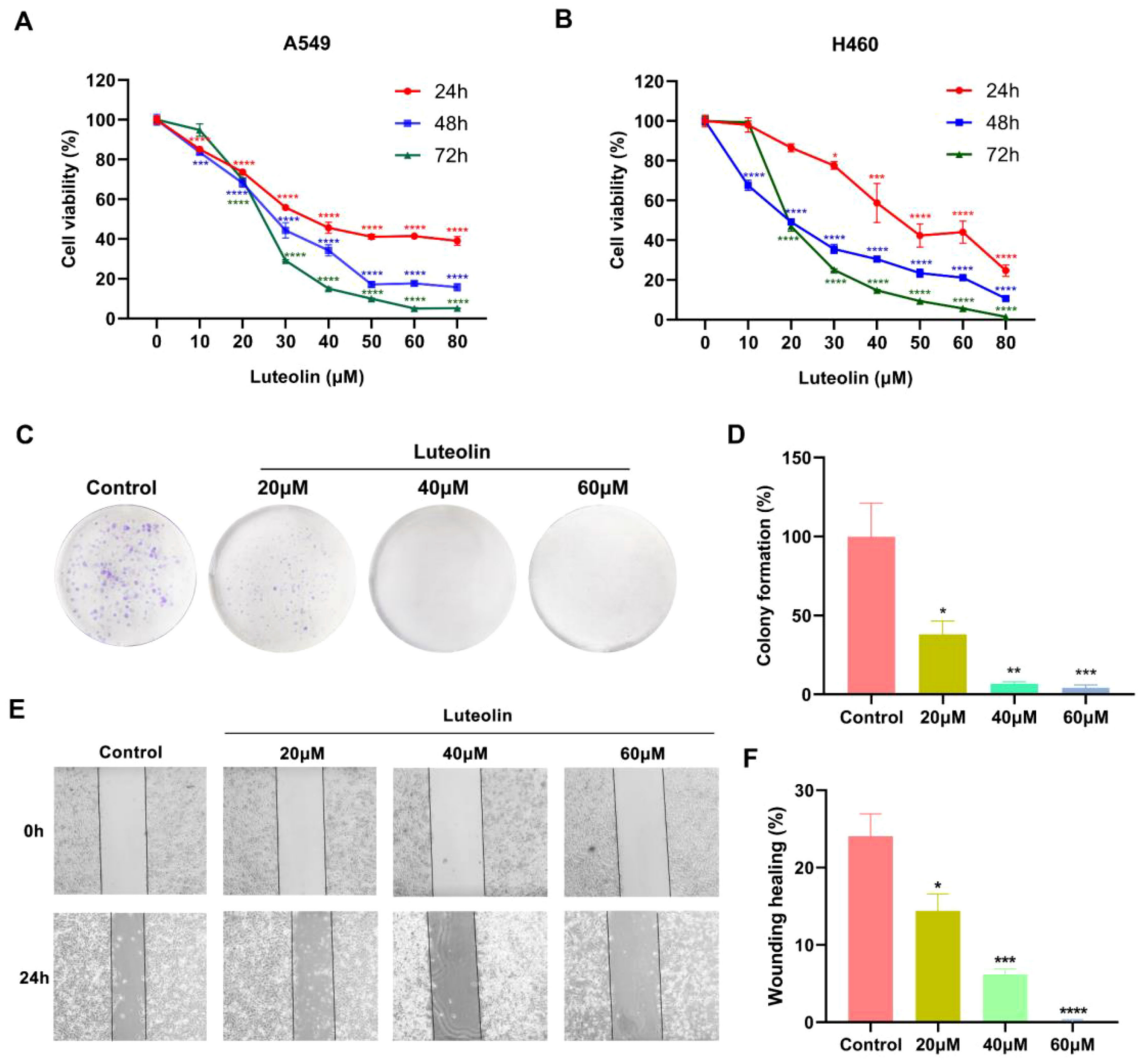
Luteolin inhibited the proliferation and migration of NSCLC cell lines. **(A, B)** The CCK8 assay demonstrated that luteolin inhibited the viability of A549 cells and H460 cells in both concentration- and time-dependent manner. **(C, D)** Colony formation assay showed that luteolin inhibited the proliferation of A549 cells. **(E, F)** Wound healing assay showed that luteolin inhibited the migration of A549 cells in a concentration-dependent manner. All data were presented as mean ± SE of three independent experiments. ^*^ *P*<0.05; ^**^ *P*<0.01; ^***^ *P*<0.001; ^****^ *P*<0.0001 compared with control group (luteolin 0 μM).

The impact of luteolin on the viability of A549 and H460 cells was assessed using a CCK8 assay. As shown in **Figures 6A, B**, luteolin significantly reduced the viability of both A549 and H460 cells in a concentration-dependent and time-dependent manner. The IC_50_ values for A549 at 24 h, 48 h, and 72 h were 41.59 μM, 27.12 μM, and 24.53 μM, respectively, while for H460 cells, they were 48.47 μM, 18.93 μM, and 20.76 μM, respectively. In the following experiments, A549 cells were selected as the experimental model and treated with luteolin at concentrations of 20 μM, 40 μM, and 60 μM. The colony formation assay indicated that luteolin significantly reduced the number of colonies formed by A549 cells (**Figures 6C, D**). These results collectively suggest that luteolin inhibits the proliferation of NSCLC cell lines.

### Luteolin inhibited the migration of A549 cells

The impact of luteolin on A549 cell migration was assessed using a wound healing assay. As shown in **Figures 6E, F**, luteolin suppressed the migration of A549 cells in a concentration-dependent manner.

### Luteolin and LY294002 induced the apoptosis of A549 cells

**Figure 7:**
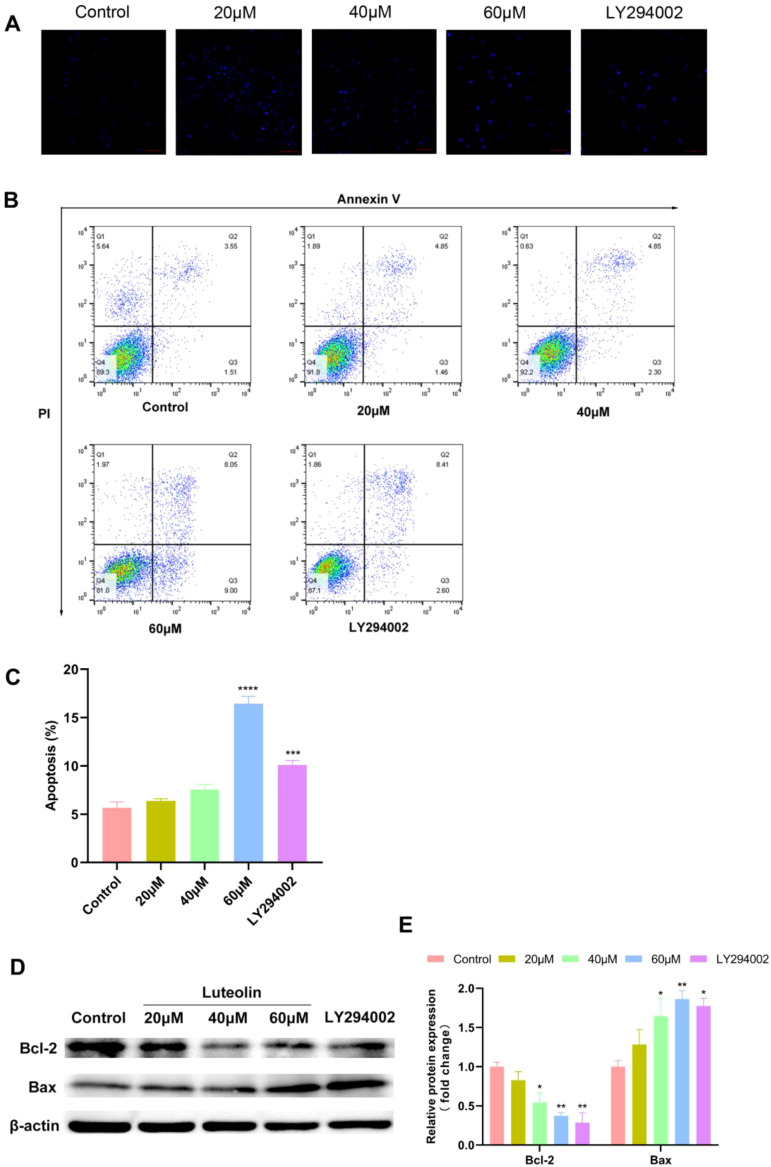
Luteolin and LY294002 induced apoptosis in A549 cells and modulated the expression of Bcl-2 and Bax. **(A–C)** The effect of luteolin and LY294002 on the apoptosis of A549 cells was detected by Hoechst 33342 staining and flow cytometry assay. The result showed that both luteolin (60 μM) and LY294002 induced the apoptosis of A549 cells. **(D, E)** Western blot analysis demonstrated that luteolin and LY294002 decreased Bcl-2 levels while increasing Bax expression. All data were presented as mean ± SE of three independent experiments. ^*^ *P*<0.05; ^**^ *P*<0.01; ^***^ *P*<0.001; ^****^ *P*<0.0001 compared with control group (luteolin 0 μM).

To investigate whether luteolin induces apoptosis in A549 cells, Hoechst 33342 staining and flow cytometry analysis were conducted. Hoechst 33342 serves as a nuclear stain displaying blue fluorescence. Under this staining, nuclei of normal cells exhibit a uniform blue, while nuclei of apoptotic cells show bright blue due to condensation and disruption (36, 37). As shown in **Figure 7A**, A549 cells treated with luteolin exhibited greater nuclear condensation compared to the control group. Flow cytometry analysis indicated that luteolin treatment enhanced the apoptosis rate in A549 cells, with pronounced effects observed in the 60μM group (**Figures 7B, C**). Additionally, western blot was employed to assess the expression of apoptosis-related proteins. The BCL-2 family, which regulates the cellular life-or-death switch and includes both pro- and anti-apoptotic members, was analyzed. As shown in **Figures 7D, E**, luteolin treatment downregulated the expression of the anti-apoptotic protein Bcl-2 and upregulated the expression of the pro-apoptotic protein Bax.

To demonstrate that the downregulation of the AKT pathway mediated apoptosis, A549 cells were treated with LY294002. LY294002 is a highly selective inhibitor of phosphatidylinositol 3 (PI3) kinase, functioning by blocking PI3K-dependent Akt phosphorylation and kinase activity (38). In this study, LY294002 (40 μM) significantly induced apoptosis in A549 cells, which corresponded with the downregulation of Bcl-2 protein expression and the upregulation of Bax expression. These results suggest that the Akt pathway is implicated in the apoptosis of A549 cells.

### Effect of luteolin and LY294002 on Akt/MDM2/p53 pathway

**Figure 8:**
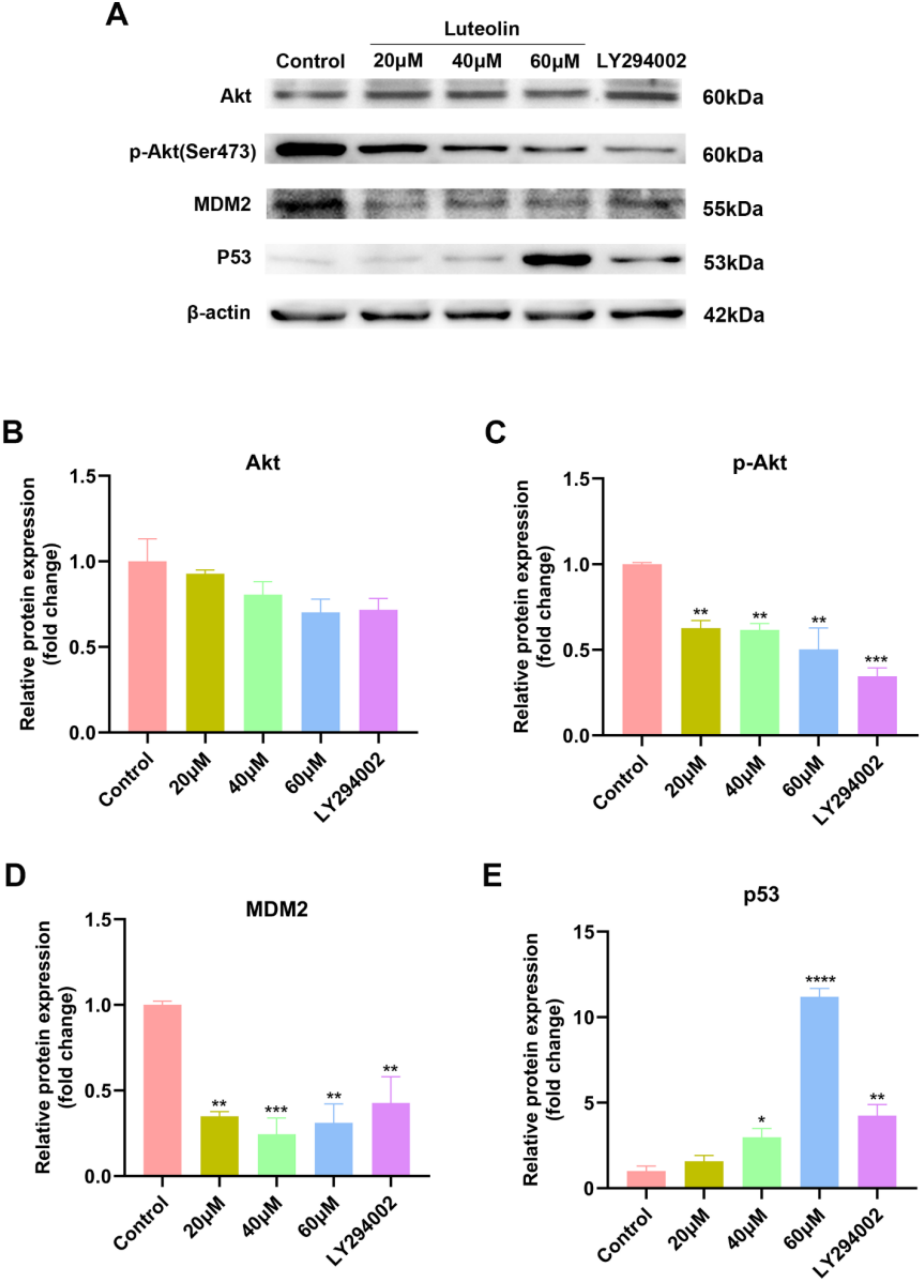
Luteolin and LY294002 decreased the expression of p-Akt (Ser473) and MDM2, while increasing the expression of p53. **(A)** Protein bands of Western blot. **(B–E)** Quantitative results of Akt, p-Akt, MDM2 and P53 normalized to β-actin. All data were presented as mean ± SE of three independent experiments. ^*^ *P*<0.05; ^**^ *P*<0.01; ^***^ *P*<0.001; ^****^ *P*<0.0001 compared with control group (luteolin 0 μM).

Based on the predicted outcomes of network pharmacology and molecular docking, it has been determined that luteolin’s therapeutic effect on NSCLC involves core targets such as TP53, EGFR, and AKT1, closely associating with the PI3K-Akt signaling pathway. Research indicates that the tumor factor mouse double minute 2 (MDM2), a downstream activating molecule of Akt, is significantly overexpressed in cancers like lung cancer and serves as a criticial negative regulator of p53 (39, 40). We hypothesized that luteolin could induce apoptosis in A549 cells via the Akt/MDM2/p53 signaling pathway. To verify the effect of luteolin and LY294002 on the AKT/MDM2/P53 pathway, western blot analysis was conducted. As shown in **Figure 8**, luteolin decreased the expression of phosphorylated Akt (Ser473) and did not significantly affect total Akt expression. Additionally, luteolin reduced MDM2 expression while increasing p53 expression, aligning with the outcomes observed following treatment with LY294002.

## Discussion

Luteolin is a natural flavonoid compound exhibiting a range of biological activities. Previous research has demonstrated that the anti-cancer properties of luteolin are related to the prevention of metabolic activation of carcinogens, induction of apoptosis, inhibition of cell proliferation, metastasis, and angiogenesis (41). To explore the multi-target and multi-pathway properties of natural compounds, network pharmacology, molecular docking, molecular dynamics simulation and *in vitro* experiments were employed to elucidate the mechanisms of luteolin in treating NSCLC.

The network pharmacology analysis indicated that there were 47 potential targets of luteolin in treating NSCLC such as TP53, AKT1, EGFR, CASP3, TNF, JUN, VEGFA, MMP1, MMP9, HMOX1, MDM2, PIK3CG, BCL2L1, etc. We then constructed a PPI network to investigate the interactions among these targets. The core targets of luteolin in treating NSCLC were identified: TP53, EGFR, AKT1, TNF, JUN, and CASP3. Molecular docking demonstrated that luteolin binds stably to these core targets. TP53, known as the guardian of the genome, is a crucial tumor suppressor, mainly acting as a transcription factor and binds to DNA to exert its anti-tumor effects (42). It enhances apoptosis by facilitating the release of downstream BCL-2 family proteins, playing a crucial role in anti-tumor therapies. However, the suppression or mutation of p53 is frequently observed in cancer. Mutant p53 not only loses its original anti-tumor activity, but also exhibits cancer-promoting effects that conntribute to tumor proliferation, invasion, metastasis, inflammation, tissue remodeling, and immune escape (43, 44). Therefore, restoring the wild-type function of p53 or increasing the activity of p53 is an effective anti-tumor strategy. AKT1 is a member of the AKT kinase family that is involved in the cell apoptosis and proliferation. Active AKT can cause tumorigenesis of a large number of human cancers, including lung, brain, gastric, colon, breast, and prostate cancer. In subsequent molecular dynamics simulations, we concentrated on the luteolin-TP53 and luteolin-AKT1 systems. The results indicated that both systems exhibited a high degree of stability.

GO enrichment analysis revealed that the biological processes and molecular functions associated with luteolin’s anti-NSCLC effects primarily encompass the negative regulation of apoptotic signaling pathways, positive regulation of phosphorylation, kinase regulatory activity, and kinase binding. Apoptosis, a type of programmed cell death, plays a crucial role in cancer progression. Induction of apoptosis in cancer cells is the most important target of many anti-cancer drugs. KEGG enrichment analysis showed that the PI3K-Akt and NF-kappa B pathways are essential for luteolin’s action on NSCLC. The PI3K-Akt signaling pathway, crucial for promoting cell survival and growth, participates in diverse cellular processes including cell cycle, growth, proliferation, survival, protein synthesis, and glucose metabolism. The dysregulation of this pathway has been implicated as a pivotal driver in 30% of cancers, with mechanisms involving activating mutations in PIK3CA, loss of function mutations in PTEN, excessive activation of upstream molecules, and gain-of-function mutations as well as amplification of AKT (45–47). Current studies have demonstrated that luteolin inhibits tumor cells by suppressing the PI3K-Akt signaling pathway. For instance, Yao et al. (48) found that luteolin inhibits the proliferation and induces apoptosis in A375 human melanoma cells by downregulating MMP-2 and MMP-9 via the PI3K-Akt pathway. Similarly, Chen et al. (49) reported that luteolin inhibits TGF-β1-induced epithelial-mesenchymal transition in lung cancer cells (A549) by interfering with the PI3K/Akt-NF-κB-Snail signaling pathway.

Akt serves as an upstream regulator of p53, playing a role in the negative regulation of p53 via the mediation on MDM2. It has been demonstrated that the phosphorylation of MDM2 on serine 166 and serine 186 by Akt is necessary for the nuclear entry of MDM2 (50). In cells containing wild-type p53, MDM2 binds directly to the p53 protein through its amino terminu, and inhibits p53 activity through following mechanisms (1): MDM2 functions as an ubiquitin ligase, promoting the degradation of p53 via the proteasome; (2) Blocking p53 from binding to target DNA; (3) Facilitating the export of p53 from the nucleus, thus impeding its role as a transcription factor (51–53). Consequently, activating wt p53 by inhibiting MDM2 represents a crucial strategy in anticancer drug development. Notably, MDM2 is the potential target of luteolin in treating NSCLC. Therefore, we hypothesized that luteolin against NSCLC via Akt/MDM2/p53 signaling pathway.

To clarify the mechanism of luteolin against NSCLC, we conducted *in vitro* experiments to validate the predictions. The p53 wild-type cell lines A549 and H460 were chosen as the experimental models. The CCK8 assay, colony formation assay and wound healing assay demonstrated that luteolin significantly inhibited the proliferation and migration of A549 cells in a concentration-dependent manner. Subsequently, we found that luteolin significantly induced apoptosis in A549 cells at a concentration of 60 μM, increased the expression of the pro-apoptotic protein Bax, and decreased the expression of the anti-apoptotic protein Bcl-2. Western blot analysis showed that luteolin downregulated the expression of p-Akt (Ser 473) and MDM2, while upregulating p53 in A549 cells. Futhermore, we also treated cells with LY294002, an AKT inhibitor, to serve as a positive control. The results indicated that the inhibition of AKT activity led to increased cell apoptosis, reduced MDM2 expression, and upregulated P53 expression. In conclusion, luteolin maight mediate apoptosis via the AKT/MDM2/P53 signaling pathway. However, whether luteolin exerts anti-NSCLC effects mainly through the AKT/MDM2/p53 signaling pathway requires further investigation.

There are several limitations of this study that need to be highlighted. First, this study was not validated *in vivo*. In addition, other signaling pathways predicted by network pharmacology, such as proteoglycans in cancers and NF-κB signaling pathway, may also contribute to luteolin’s antitumor effects in NSCLC. This warrants further investigation.

## Conclusion

In this study, we investigated the effects and mechanisms of luteolin on NSCLC using network pharmacology and *in vitro* experiments. The findings indicated that luteolin could against NSCLC by inhibiting cell proliferation and migration, and by inducing apoptosis in A549 cells. The pro-apoptotic effect of luteolin may be associated with the modulation of the Akt/MDM2/p53 signaling pathway.

## Data Availability

The datasets used and/or analyzed during the current study are available from the corresponding author on reasonable request.
